# Genetic parameters for growth and faecal worm egg count following *Haemonchus contortus* experimental infestations using pedigree and molecular information

**DOI:** 10.1186/1297-9686-46-13

**Published:** 2014-02-14

**Authors:** Fabrizio Assenza, Jean-Michel Elsen, Andrés Legarra, Clément Carré, Guillaume Sallé, Christèle Robert-Granié, Carole R Moreno

**Affiliations:** 1 INRA, UR0631 Station d'Amélioration Génétique des Animaux, F-31326 Castanet-Tolosan, France; 2 INRA, UMR1282 Infectiologie et Santé Publique, F-37380 Nouzilly, France; 3 Université François Rabelais de Tours, UMR1282 Infectiologie et Santé Publique, F-37000 Tours, France

## Abstract

**Background:**

Haemonchosis is a parasitic disease that causes severe economic losses in sheep industry. In recent years, the increasing resistance of the parasite to anthelmintics has raised the need for alternative control strategies. Genetic selection is a promising alternative but its efficacy depends on the availability of genetic variation and on the occurrence of favourable genetic correlations between the traits included in the breeding goal. The objective of this study was twofold. First, to estimate both the heritability of and the genetic correlations between growth traits and parasite resistance traits, using bivariate linear mixed animal models, from the phenotypes and genotypes of 1004 backcross lambs (considered as a single population), which underwent two subsequent experimental infestations protocols with *Haemonchus contortus*. Second, to compare the precision of the estimates when using two different relationship matrices: including pedigree information only or including also SNP (single nucleotide polymorphism) information.

**Results:**

Heritabilities were low for average daily gain before infestation (0.10 to 0.15) and average daily gain during the first infestation (0.11 to 0.16), moderate for faecal egg counts during the first infestation (0.21 to 0.38) and faecal egg counts during the second infestation (0.48 to 0.55). Genetic correlations between both growth traits and faecal egg count during the naïve infestation were equal to zero but the genetic correlation between faecal egg count during the second infestation and growth was positive in a *Haemonchus contortus* free environment and negative in a contaminated environment. The standard errors of the estimates obtained by including SNP information were smaller than those obtained by including pedigree information only.

**Conclusions:**

The genetic parameters estimates suggest that growth performance can be selected for independently of selection on resistance to naïve infestation. Selection for increased growth in a non-contaminated environment could lead to more susceptible animals with long-term exposure to the infestation but it could be possible to select for increased growth in a contaminated environment while also increasing resistance to the long-term exposure to the parasite. The use of molecular information increases the precision of the estimates.

## Background

*Haemonchus contortus* (*H. contortus)* is a nematode that feeds on blood through the abomasal mucosa of bovine, ovine and caprine species [1]. The cost of *H. contortus* infection or haemonchosis for the production sectors of sheep farming in terms of anthelmintic treatments that are currently the most popular control strategy, and the resulting economic loss have been estimated in different countries to be in the order of several million dollars per year [2-7]. Furthermore, anthelmintics tend to select the parasite population under treatment for resistance to the anthelmintic itself [8,9], which increases the cost of haemonchosis even more.

Both the long-term loss of efficacy and the growing public concern for the use of chemicals in food production fostered the research on alternative control strategies or combinations of them [10], among which genetic selection is one of the most promising approaches [11-25]. Simulation studies based on evolutionary genetics [26] predict a breeding plan’s long-term outcomes and also the efficacy of genetic selection as a control strategy. Since genetic improvement depends on the genetic parameters of the traits under selection, the estimates of these parameters must be as precise as possible for reliable long-term predictions. However, consistent estimates of the genetic correlation between production traits and parasite resistance traits have not been reported in the literature [27-33]. Since most of the estimates found in the literature are computed from observations in natural conditions, where it is not possible to precisely define neither the nutritional level of the diet nor the larval challenge on the pasture, the reason why no consistent estimates are available may be due to the interaction between these two factors [34].

The first objective of this study was to estimate the heritability of average daily gain and faecal egg count from experimental observations, together with the genetic correlations between them. We report the results of an analysis performed on 1004 phenotypic records of growth traits and faecal egg counts collected on genotyped (50 k SNPchip) back-cross lambs (25% Martinique black belly and 75% Romane), following two experimental infestations with *H. contortus.* The genetic parameters have been estimated both by using pedigree information only and pedigree and SNP (single nucleotide polymorphism) chip information jointly. Computing the relatedness between individuals using pedigree information only is based on expectation and results in an estimate corresponding to the average number of alleles shared by two individuals, for example: all the individuals belonging to the same full-sibs group would have a coefficient of 0.5 between each other, which means that it does not take into account the deviation from this average caused by segregation and recombination. However, including molecular information makes it possible to compute the relatedness between individuals by identifying on a relatively dense map the actual number of alleles they share, which provides a more detailed estimate of the relatedness between individuals [35]. Since the observations used in this study were collected on four large groups of half-sibs, the second objective of the study was to test whether including SNP information could help reach more precise estimates than using pedigree information only when the pedigree of the population is poorly informative. Although previous studies have already explored the amount of genetic variability for parasite resistance traits, this study features several novelties: the genetic parameters reported here are estimated from phenotypes collected in experimental settings rather than natural infestation, the growth traits analysed are the average daily gains before infestation and during infestation rather than the body weights, and finally the estimates reported here also feature molecular information rather than pedigree information only.

## Methods

### Experimental design

**Figure 1:**
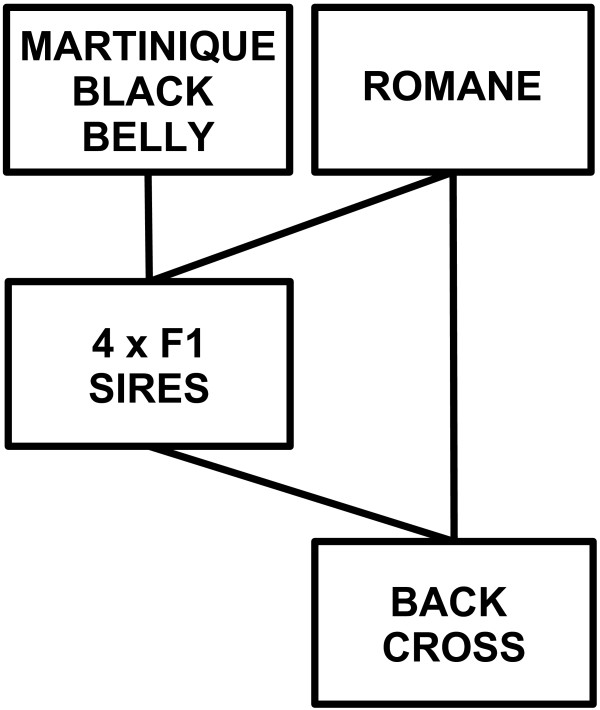
**Schematic representation of the pedigree structure.** MBB: Martinique Black Belly; ROM: Romane; FI are the four F1 sires resulting from the cross between MBB sires and ROM dams; BC is the back cross population obtained by mating the F1 sires to ROM dams.

The population in which the observations were collected from resulted from a back-cross mating scheme between two pure-bred populations: Martinique Black Belly (MBB) and Romane (ROM). MBB is a tropical sheep breed, which is characterized by adaptation to heat-stress, to parasitism and to extensive raising conditions. The ROM sheep breed features good productive performances (both for meat production and prolificacy) and no selection for resistance to parasites. The pedigree used in the analysis (Figure 1) was three generations deep and counted a total of 3164 animals. Four F1 sires were produced by crossing MBB and ROM individuals. The sires were mated by intra-uterine artificial insemination to 829 pure-bred ROM dams in order to obtain 1265 back-cross offspring (BC), the number of animals used in this study from each group of half-sibs were 282, 251, 247 and 223, respectively. All animals were managed as a commercial flock, the experimental protocol complied to the European ethical policy and was approved by the ethical committee “comite d’ethique CIRAD-INRA”.

### Genotypes

A total of 1044 animals among the population of the back-cross lambs and their four F1 sires were genotyped with the OvineSNP50 Beadchip (Illumina Inc., San Diego, CA). Quality control of the SNPs included the following tests: (1) 50 animals were genotyped twice in order to assess the technical reliability of the genotyping, which resulted in a value of 99.9%, (2) individuals with a call rate below 98% and SNPs with a call rate below 97% or with a minor allele frequency below 1% or featuring a deviation from expected heterozygosity or showing mendelian inconsistencies were discarded (p < 10^-6^) and (3) quality control of the genotypes resulted in 42 469 SNPs that comply with all quality checks. More details about the genomic information can be found in Sallé et al. [36].

### Phenotypes

Phenotypes on growth traits and parasite resistance traits were collected on the BC animals only by performing the following experimental protocol. The lambs were weaned around 64 or 45 days, depending whether their mother was either primiparous or not, and grew in a *H. contortus* free environment until the first experimental infestation was performed. During this period the animals were kept in an *H. contortus* free environment and were weighed twice: at weaning and at the end of the growing period. The growing period ended with the beginning of the following experimental infestation protocol, which also determined when the environment was to be contaminated with *H. contortus* larvae: at around 90 days of age, the lambs received an oral inoculation of 10 000 L3 larvae of *H. contortus* (ENVT strain [37]) and around 41 days after the infestation, they received an anthelmintic treatment (LEVAMISOLE 5%, Vibrac S.A., Carros, France, 7.5 mg/kg live weight). During the infestation, two faecal samples were collected, at 25 and 35 days after infestation, and the animals were weighed on the day of treatment. Then, they entered a recovery period of 8 days, at the end of which they were infested again with the same infestation protocol. During the second infestation, two faecal samples were collected as before but animals were not weighed. During the whole protocol, the animals were fed ad libitum on a diet that covered largely their requirements. The faecal egg count in each sample was measured by a modified McMaster procedure [38]. The average of the two faecal egg count observations was computed for each infestation. The latter values were transformed by taking their fourth root in order to bring their distribution closer to normality. A further transformation was applied in order to scale the standard deviation to 1 and avoid zero values. The variables obtained were called: FEC1 (transformed faecal egg counts during the first infestation) and FEC2 (transformed faecal egg counts during the second infestation). The average daily gain from weaning to infestation (ADG0) and average daily gain during the first infestation (ADG1) were computed as follows: $\mathrm{ADG}0=\frac{\mathrm{growth}0}{\mathrm{time}0}\;\mathrm{and}\;\mathrm{ADG}1=\frac{\mathrm{growth}1}{\mathrm{time}1}$, where *growth0* and *time0* are the weight gain and the days running from the weaning day to the day of beginning of the first infestation, respectively; *growth1* and *time1* are the weight gain and the days running from the first day of the first infestation to the day of treatment of the first infestation, respectively.

A transformation for scaling the standard deviation to 1 and avoiding 0 s was applied on growth traits as well. The observations outside a range of 2.96 standard deviations around the average of each trait were considered atypical and excluded from the analysis. Finally, only the animals featuring a valid observation both on genotyping and on at least one trait were included in the analysis, which resulted in 40 animals being discarded and 1004 animals being included.

### Statistical analysis

Estimation of the genetic parameters was performed by considering the back-cross population as a single breed population. The breed proportions are taken into account by the genetic relationship matrix in the model including SNP information but could not be taken into account in the model including pedigree information only due to convergence failure. The heritability of each trait and both the genetic correlation and phenotypic correlation between each pair of traits were estimated by bivariate animal mixed models, which were solved by the AIREML procedure implemented in AIRemlF90 software [39]. This software features by default the correction for the change in the definition of the base population so that the estimates obtained when using pedigree information only were comparable to those obtained when including molecular information [40,41]. The bivariate mixed model reads as follows:

$$\left[\begin{array}{c} y_{1}\\ y_{2} \end{array}\right]=\left[\begin{array}{cc} X_{1} & 0\\ 0 & X_{2} \end{array}\right]\left[\begin{array}{c} b_{1}\\ b_{2} \end{array}\right]+\left[\begin{array}{cc} Z_{1} & 0\\ 0 & Z_{2} \end{array}\right]\left[\begin{array}{c} a_{1}\\ a_{2} \end{array}\right]+\left[\begin{array}{c} e_{1}\\ e_{2} \end{array}\right],$$

where **y**_**1**_ and **y**_**2**_ are the vectors of observations of trait one and two, respectively, **X**_**1**_ and **X**_**2**_ are incidence matrices relating each observation to its respective set of fixed effects and **b**_**1**_ and **b**_**2**_ are the vectors of the fixed effects: weight at weaning (for ADG0 only) or weight at first infestation (for all the other traits), contemporary group (identified by year, season, weighting lot and infestation lot), sex and feeding mode (breast feeding or bottle feeding). **a**_**1**_ and **a**_**2**_ are the vectors of random animals breeding values, with the associated incidence matrices **Z**_**1**_ and **Z**_**2**_. **e**_**1**_ and **e**_**2**_ are the vectors of random residuals. It is assumed that the random effects are normally distributed and feature the following variance-covariance structure:

$$V\left[\begin{array}{l} a_{1}\\ a_{2}\\ e_{1}\\ e_{2} \end{array}\right]=\left[\begin{array}{cccc} \sigma_{g11}^{2}T & & \mathrm{symmetric} & \\ \sigma_{g21}T & \sigma_{g22}^{2}T & & \\ 0 & 0 & \sigma_{e11}^{2}I & \\ 0 & 0 & \sigma_{e21}I & \sigma_{e22}^{2}I \end{array}\right],$$

where $\sigma_{g11}^{2},\;\sigma_{g22}^{2}\;\mathrm{and}\;\sigma_{g21}$ are the genetic variances and the genetic covariance between traits 1 and 2, $\sigma_{e11}^{2},\;\sigma_{e22}^{2}\;\mathrm{and}\;\sigma_{e21}$ are the residuals variances and the residuals covariance between traits 1 and 2, **I** is an identity matrix and **T** is the genetic relationship matrix between the animals.

The genetic and phenotypic correlations between each couple of traits were computed using two different genetic relationship matrices: one computed by using pedigree information only (PED model) and one computed by including both SNP and pedigree information jointly (SNPED model). The **T** matrix used in the PED model was computed according to Quaas [42] and without using molecular information. The **T** matrix used in the SNPED model was computed according to VanRaden [43], using a three-generation deep pedigree and the following weights: **T** = 0.95**G** + 0.05**A**_**22**_, where **A**_**22**_ is the relationship matrix between the genotyped animals computed by using the pedigree information only [42] and **G** is the genomic relationship matrix among genotyped animals. In the software package used for this study [39], the **G** matrix is computed by default as follows: **G** = **WK**^− 1^**W**, [43] where **W** is a rectangular matrix (number of animals by number of SNPs’ alleles) with elements: *w*_*ij*_ = *f*_*ij*_ − 2p_*j*_, where *f*_*ij*_ is a scalar equal to the number of copies of one allele an animal i has at locus j, p_j_ is the frequency of allele j in the population, **K** is the diagonal matrix of the scaling parameters with elements: K_*jj*_ = 2 ∑ *p*_*j*_(1 − *p*_*j*_). The weights of **G** and are used for bending the genetic relationship matrix and make it positive definite, as required for its inversion [43], this is similar to the bending procedure occurring in AIREML algorithms for keeping the variance covariance matrix positive definite [42].

The precision of the heritability and correlation estimates was computed by estimating their standard error according to the following formulas [44]:

$$\mathrm{SE}\left(h_{i}^{2}\right)=\sqrt{h_{i}^{4}\left(\frac{V\left(\sigma_{\mathrm{gii}}^{2}\right)}{{\left(\sigma_{\mathrm{gii}}^{2}\right)}^{2}}+\frac{V\left(\sigma_{\mathrm{pii}}^{2}\right)}{{\left(\sigma_{\mathrm{pii}}^{2}\right)}^{2}}-\frac{2C\left(\sigma_{\mathrm{gii}}^{2},\sigma_{\mathrm{pii}}^{2}\right)}{\sigma_{\mathrm{gii}}^{2}\sigma_{\mathrm{pii}}^{2}}\right)},$$

where $\mathrm{SE}\left(h_{i}^{2}\right)$ is the standard error of the estimate of the heritability of trait i, $\sigma_{\mathrm{gii}}^{2}$ and $\sigma_{\mathrm{pii}}^{2}$ are the estimates of its genetic and phenotypic variances, respectively, $V\left(\sigma_{\mathrm{gii}}^{2}\right)$, $V\left(\sigma_{\mathrm{pii}}^{2}\right)$ and $C\left(\sigma_{\mathrm{gii}}^{2},\sigma_{\mathrm{pii}}^{2}\right)$ are the variances of the estimated values and the covariance between the estimated values, respectively, obtained from the information matrix [44]:

$$\begin{array}{c} \mathrm{SE}\left(r_{g}\right)=\sqrt{r_{g}^{2}\left(\frac{V\left(\sigma_{g11}^{2}\right)}{4{\left(\sigma_{g11}^{2}\right)}^{2}}+\frac{V\left(\sigma_{g22}^{2}\right)}{4{\left(\sigma_{g22}^{2}\right)}^{2}}+\frac{V\left(\sigma_{g21}\right)}{{\left(\sigma_{g21}\right)}^{2}}\right)}+\\ \sqrt{r_{g}^{2}\left(\frac{C\left(\sigma_{g11}^{2},\sigma_{g22}^{2}\right)}{2\sigma_{g11}^{2}\sigma_{g22}^{2}}-\frac{C\left(\sigma_{g11}^{2},\sigma_{g21}\right)}{\sigma_{g11}^{2}\sigma_{g21}}-\frac{C\left(\sigma_{g22}^{2},\sigma_{g21}\right)}{\sigma_{g22}^{2}\sigma_{g21}}\right)} \end{array},$$

where *SE*(*r*_*g*_) is the standard error of the estimate of the genetic correlation, *r*_*g*_ is the estimated value of the genetic correlation; are $\sigma_{g11}^{2},\;\sigma_{g22}^{2}\;\mathrm{and}\;\sigma_{g21}$ the estimates of the genetic variance components described above and V(.) and C(..) are the variance of the estimates between parenthesis and the covariance between the estimates between parenthesis, respectively. The same formula was used to compute the standard error of the phenotypic correlation, but by filling in the entries concerning phenotypic variances and covariances.

### Significance tests

The parameters under study are the ratio of two normally distributed variables (heritability) and the ratio between a gaussian variable over the square root of the product of two gaussian variables (correlations). The sampling distribution of heritability can be approximated to a gaussian distribution under certain specific conditions only [45]. When these conditions are filled, the significance test for gaussian variables can be applied. However, the significance tests for the correlation coefficient can be developed by deriving its confidence interval according to Fisher's Z-transformation [46]. Otherwise, both parameters can be tested by using a re-sampling procedure such as delete-d jackknife: where d is the number of observations randomly discarded from the dataset and n^1/2^ < d < n (n is the total number of observations in the dataset) [47]. One thousand sub-samples of the whole dataset were created by randomly discarding 20% of the observations. Each parameter computed above was re-estimated from each sub-sample in order to build its empirical distribution. The empirical distribution of each parameter was used to compute the confidence interval of each parameter by taking its 2.5% quantile as the lower bound and its 97.5% quantile as the upper bound of each estimate. The null hypothesis “the estimate is not different from 0” was tested as follows: if the confidence interval of the estimate included 0, then the null hypothesis was not rejected, otherwise the alternative hypothesis “the estimate is different from 0” was accepted. In order to test whether the SNPED and SNP models converged to the same estimate, the distribution of the difference between the PED and SNPED estimates was built for each estimate as follows:

$$\left[d_{i}\right]=\left[\mathrm{Pe}d_{i}‒\mathrm{Snpe}d_{i}\right],$$

where **Ped**_**i**_ is the vector containing the n realizations of the estimate obtained from the PED model, **Snped**_**i**_ is the vector containing the n realizations of the estimate obtained from the SNPED model, **d**_**i**_ the vector of the differences between and each element of **Ped**_**i**_ and **Snped**_**i**_. The confidence interval of the distribution of the difference was computed as above. The null hypothesis “the difference between the estimate obtained from the PED model and the estimate obtained from the SNPED model is 0” was tested against the alternative hypothesis “the difference between the estimate obtained from the PED model and the estimate obtained from the SNPED model is not 0” as above as well.

## Results and discussion

### Phenotypic variation

Table 1 shows the summary statistics of the variables analysed. The transformations applied to the raw faecal egg counts resulted in the profile of their distribution being closer to normality. The skewness and normalized kurtosis of FEC1 changed from 2.11 to −0.44 and from 6.56 to 0.53, respectively; the skewness and normalized kurtosis of FEC2 changed from 3.87 to 0.14 and from 23.92 to −0.74, respectively. The number of observations on each trait together with the average, standard deviation, minimum and maximum of the raw observations are in Table 1. ADG1 was significantly lower than ADG0 (p_value < 0.0001), indicating that infested animals had a slower growth than the parasite-free animals, as expected due to the infestation [1].

**Table 1:** Descriptive statistics of the raw observations

**Raw observations**	**Number of observations**	**Average**	**Standard deviation**	**Min**	**Max**
ADG0_RAW_ (g/day)	997	293.3	69.4	93.0	504.5
ADG1_RAW_ (g/day)	963	102.8	43.3	−27.0	230.0
FEC1_RAW_ (eggs/g)	987	10494	9827	0	75898
FEC2_RAW_ (eggs/g)	967	2724	4259	0	42667

**ADG0**_**RAW**_ = average daily gain before infestation; **ADG1**_**RAW**_ = average daily gain during the first infestation; **FEC1**_**RAW**_ = faecal egg count during the first infestation; **FEC2**_**RAW**_ = faecal egg count during the second infestation.

Table 2 shows the estimate of the phenotypic correlations (below the diagonal) obtained from the two models for each pair of traits. Although the SNPED and PED models did not always converge on the same value, according to the significance tests described above, these estimates were not significantly different between the two models. The estimates of the phenotypic correlations between ADG0 and both FEC1 and FEC2 were not significantly different from 0: -0.01 (SE = 0.15) and 0.01 (SE = 0.18) for the PED model and 0.02 (SE = 0.11) and 0.04 (SE = 0.11) for the SNPED model. These results suggest that the phenotype for growth rate in a *H. contortus* free environment was unrelated to the parasite resistance phenotype. However, the estimates of both the phenotypic correlations between ADG1 and FEC1 and between ADG1 and FEC2 were negative: -0.24 (SE = 0.15) and −0.20 (SE = 0.19) for the PED model and −0.23 (SE = 0.11) and −0.19 (SE = 0.11) for the SNPED model. These results suggest an inverse proportionality between the growth rate and the parasite burden, in accordance with the finding that contaminated animals had a slower growth than non-contaminated animals. The average faecal egg count during the second infestation was significantly lower than the faecal egg count during the first infestation (p_value < 0.0001), which suggests that the development of a specific immune response was triggered by the first infestation, that enhanced the intrinsic resistance of the animals to subsequent infestations [48]. Furthermore, the positive estimate of the phenotypic correlation between FEC1 and FEC2, 0.46 (SE = 0.43) for the PED model and 0.62 (SE = 0.20) for the SNPED model, shows that the animals featuring higher (or lower) than average FEC1 are likely to express higher (or lower) than average FEC2, and vice versa. This suggests that a repeatable variation in susceptibility occurs within the population.

**Table 2:** Heritabilities, phenotypic correlations and genetic correlations obtained by the SNPED model and the PED model

**Traits**	**Model**	**ADG0**	**ADG1**	**FEC1**	**FEC2**
ADG0	**SNPED**	0.15 (0.07)^*^	-	0.11 (0.47)	0.57 (0.38)^*^
	**PED**	0.10 (0.08)^*^	-	−0.52 (1.06)	0.25 (0.85)
ADG1	**SNPED**	-	0.11 (0.06)^*^	−0.12 (0.58)	−0.54 (0.53)^*^
	**PED**	-	0.16 (0.04)^*^	−0.19 (0.80)	−0.48 (0.67)^*^
FEC1	**SNPED**	0.02 (0.11)	−0.23 (0.11)^*^	0.38 (0.04)^*^	0.62 (0.20)^*^
	**PED**	−0.01 (0.15)	−0.24 (0.15)^*^	0.21 (0.06)^*^	0.46 (0.43)^*^
FEC2	**SNPED**	0.04 (0.11)	−0.19 (0.11)^*^	0.31 (0.08)^*^	0.48 (0.06)^*^
	**PED**	0.01 (0.18)	−0.20 (0.19)^*^	0.29 (0.14)^*^	0.55 (0.09)^*^

**ADG0** = average daily gain before infestation; **ADG1 =** average daily gain during the first infestation; **FEC1** = faecal egg count during the first infestation; **FEC2** = faecal egg count during the second infestation; **SNPED** refers to estimates obtained by using the joint pedigree and molecular information relationship matrix (SNPED model); **PED** refers to the pedigree-only relationship matrix (PED model); the correlations between **ADG0** and **ADG1** were much more sensitive than others to the starting values used for the estimation and to re-sampling, and are not presented; **heritabilities** are on the block diagonal, **genetic correlations** are above the diagonal and **phenotypic correlations** are below the diagonal; **standard errors** of the estimates are between brackets; superscript ^*^ marks the estimates that were significantly different from 0.

### Genetic variation

Table 2 summarizes the estimates of the heritabilities of each trait (block diagonal) and the estimates of the genetic correlations (above the diagonal) between each pair of traits obtained with the PED and SNPED models, together with the standard error of each estimate (between brackets). Due to the pedigree structure that includes only four sires, both the standard errors and the 95% confidence intervals of the estimates were indeed large (in particular, those of the PED model), which led to no significant difference between the estimates obtained from the two models. The results obtained were in general coherent between models, except for the genetic correlation between ADG0 and FEC1, which was positive with the SNPED model and negative with the PED model. However, the latter estimate had a standard error as large as half the parameter space, which resulted in the confidence of the difference between the two estimates to include 0. The reason for this possible inconsistency cannot be defined by the data available for this study. We can only speculate that it could be the result of the segregation variance captured by the SNP chip, because the phenotypes analysed were collected on the back-cross offspring of only four sires. This pedigree structure is indeed poorly informative if the estimate is computed by using pedigree information only, which is clearly shown by the huge standard error obtained with the PED model. However, this does not explain why such a big difference in the estimates occurs between some pairs of traits only. The heritability of ADG0 was low for both models, 0.10 (SE = 0.08) for the PED model and 0.15 (SE = 0.07) for the SNPED model, and is close to the value 0.17 found in the review of Safari et al. [27]. The heritability of ADG1 was low as well, 0.16 (SE = 0.04) and 0.11 (SE = 0.06) for the PED and the SNPED model respectively and no estimates were found in the literature for ADG1. The estimates for the heritabilities of both faecal egg counts were found higher than those obtained in previous studies [29-32]: FEC1 was 0.21 (SE = 0.06) and 0.38 (SE = 0.04) for the PED and the SNPED model, respectively; FEC2 was 0.55 (SE = 0.09) and 0.48 (SE = 0.06) for the PED and the SNPED model, respectively. The reason for finding a higher value than in previous studies could be twofold. First, it could be due to the fact that most of the estimates found in the literature are computed from observations in natural rather than experimental conditions, the latter of which allows controlling more strictly the environmental conditions and hence could reduce phenotypic variation. The second reason could be the occurrence of breed specific alleles that segregate within the back-cross population, which inflate the genetic variance compared to a pure breed population. These values confirm the availability of a moderate genetic potential in sheep that could be exploited to enhance resistance to parasites.

Concerning previous estimates of the genetic correlation between growth traits and faecal egg counts, no other estimations of these parameters based on experimental infestation were found in the literature. Furthermore, previous studies on similar traits based on natural infestations do not show consistency among them [29-32], which could be explained by the uncontrolled variation in the larval challenge, in the pathogenicity of the parasite in each population, in the feed intake and the interaction between these three factors [34].

Concerning the genetic correlations between growth traits (ADG0 and ADG1) and parasite resistance during the naïve infestation (FEC1), the following picture can be drawn. In accordance with the estimates of the phenotypic correlation between ADG0 and FEC1, the genetic correlations between these traits were also not significantly different from 0: -0.52 (SE = 1.06) for the PED model and 0.11 (SE = 0.47) for the SNPED model. The same results were obtained with the estimate of the genetic correlations between ADG1 and FEC1 (although their phenotypic correlations were negative according to both models): -0.19 (SE = 0.80) for the PED model and −0.12 (SE = 0.58) for the SNPED model. These results suggest that during the naïve infestation, the genotype for growth (ADG0 and ADG1) could be expressed independently from the genotype for parasite resistance (FEC1).

The results obtained for the genetic correlations between growth traits and the long-term resistance (FEC2) were on the contrary significantly different from 0. On the one hand, the genetic correlation between ADG0 and FEC2 was positive but not significantly different from 0 according to the PED model (0.25, SE = 0.85) and positive (0.57, SE = 0.38) according to the SNPED model. According to the approximate standard errors, the positive estimate obtained with the SNPED model is more reliable and suggests that if animals were selected for growth in a parasite-free environment, a correlated selection response for lower long-term resistance to gastrointestinal parasites could occur as well. This estimate supports the hypothesis that enhancing growth traits could come to a cost to the sheep's long-term susceptibility to parasite infestations, and vice versa [49,50]. On the other hand, the correlation between ADG1 and FEC2 was consistently negative between models: -0.48 (SE = 0.67) for the PED model and −0.54 (SE = 0.53) for the SNPED model, which suggests that growth during the naïve infestation can be enhanced together with long-term resistance to the infestation within a single purebred line.

The genetic correlation between FEC1 and FEC2 was 0.46 (SE = 0.43) and 0.62 (SE = 0.20) for the PED and SNPED models, respectively, which suggests that these traits have different determinisms. While FEC1 represents a measure of the parasite resistance expressed by a naïve lamb, FEC2 is a measure of the parasite resistance expressed by an immunized lamb, and indeed the mechanisms by which these types of animals respond to the infestation are different [48].

The estimate of genetic correlation between ADG0 and ADG1 was unstable due to its sensitivity to the starting values used for its estimation and is not reported.

### Standard errors

Table 3 shows both the ratio of the standard errors of the estimates obtained from the PED model over the standards error obtained from the SNPED model, which ranged from 1.04 to 2.25 and also the ratio between the width of the confidence intervals of the estimates obtained from the PED model over the width of the confidence intervals obtained from the SNPED model, which spanned an interval between 0.93 and 4.21. According to the ratio of the standard errors, the SNPED model always converged to more precise values, while, according to the ratio of the width of the confidence intervals, the estimate of the phenotypic correlation between growth traits and FEC1 obtained from the PED model was slightly more precise.

**Table 3:** Ratios between the precision estimators for the PED model and for the SNPED model

**Traits**	**ADG0**	**ADG1**	**FEC1**	**FEC2**
**ADG0**	^SE^1.12	-	^SE^2.24	^SE^2.25
	^CI^1.42		^CI^1.26	^CI^4.21
**ADG1**	-	^SE^1.23	^SE^1.38	^SE^1.26
		^CI^2.37	^CI^1.30	^CI^1.57
**FEC1**	^SE^1.37	^SE^1.33	^SE^1.72	^SE^2.22
	^CI^0.93	^CI^0.98	^CI^1.5	^CI^2.2
**FEC2**	^SE^1.73	^SE^1.68	^SE^1.76	^SE^1.67
	^CI^1.41	^CI^1.30	^CI^1.25	^CI^1.9

**ADG0** = average daily gain before infestation; **ADG1** = average daily gain during the first infestation; **FEC1** = faecal egg count during the first infestation; **FEC2** = faecal egg count during the second infestation; the table shows both values of the ratio between the approximate standard errors (marked with superscript SE) and the ratios of the width of the confidence intervals obtained from the empirical distribution (marked with superscript CI) of the parameters estimates obtained by using either pedigree relationship matrix or the joint pedigree and molecular information relationship matrix; the ratios on the diagonal refer to the **heritability** estimates; the ratios above the diagonal refer to the **genetic correlation** estimates; the ratios below the diagonal refer to the **phenotypic correlation** estimates; the correlations between **ADG0** and **ADG1** were much more sensitive than others to the starting values used for the estimation and to re-sampling, and are not presented.

The results obtained show that including SNP information in the computation of the relationship matrix between individuals can increase the precision of the genetic parameter estimates up to twice the precision obtained by using pedigree information only [51]. The increase in precision can be explained by the fact that SNP information allows to compute more precisely than pedigree information what proportion of genome two individuals actually have in common. The pedigree structure in the data available for this study was not ideal to estimate genetic parameters by pedigree information only because all observations are recorded on a population of animals composed of four groups of half-sibs. Such a structure causes the pedigree-based relationship matrix to predict that within each group of half-sibs all animals share one quarter of the sire’s genome. Whereas, the marker-based relationship matrix allows capturing the segregation variance, which means capturing the random deviation of the proportion of genes shared by two individuals around the expected proportion of shared genes according to the pedigree [35].

## Conclusions

According to the results obtained by the model including both pedigree and molecular information, the genotypes for growth and for resistance to naïve infestation can be selected for independently. However, the genetic correlations between long-term parasite resistance traits and growth traits were different from 0 and suggest that increasing growth performance in a *H. contortus* free environment could result in more susceptible animals, whereas growth performance in a contaminated environment can be increased while enhancing long-term resistance to *H. contortus*. The two results taken together can also be interpreted as an indication of genotype by environment interaction affecting growth expressed across the two environments [52]. The model that includes pedigree information only converged to similar results, except for the genetic correlation between growth before infestation and faecal egg count during the first infestation which was affected by a very large standard error. The reason for this inconsistency needs further investigation.

This study shows that, when the pedigree is poorly informative using molecular information and pedigree information jointly result in more precise genetic parameters than using pedigree only.

## Competing interests

The authors declare that they have no competing interests.

## Authors’ contributions

FA edited the observations on average daily gain, estimated the genetic parameters and their standard errors, interpreted the results and wrote the manuscript. CR supervised the PhD project, helped with performing the statistical analysis and with the interpretation of its results and revised the manuscript. JME, AL and CRG helped with performing the statistical analysis, interpreting its results and revising the manuscript. CC programmed the Jackknife procedure in parallel and helped with the interpretation of the results and manuscript review. GS performed the data editing of both the observations on faecal egg counts and of SNP data, of results interpretation and manuscript review. All authors read and approved the final manuscript.
